# Vertical Microfluidic Trapping System for Capturing and Simultaneous Electrochemical Detection of Cells †

**DOI:** 10.3390/s24206638

**Published:** 2024-10-15

**Authors:** Lilia Bató, Péter Fürjes

**Affiliations:** 1 Microsystems Lab, Institute of Technical Physics and Materials Science, HUN-REN Centre for Energy Research, H-1121 Budapest, Hungary; furjes.peter@ek.hun-ren.hu; 2 Doctoral School on Materials Sciences and Technologies, Óbuda University, H-1034 Budapest, Hungary

**Keywords:** microfluidics, electrode array, EIS, cell trapping

## Abstract

Electrochemical impedance spectroscopy (EIS) is a non-invasive and label-free method widely used for characterizing cell cultures and monitoring their structure, behavior, proliferation and viability. Microfluidic systems are often used in combination with EIS methods utilizing small dimensions, controllable physicochemical microenvironments and offering rapid real-time measurements. In this work, an electrode array capable of conducting EIS measurements was integrated into a multichannel microfluidic chip which is able to trap individual cells or cell populations in specially designed channels comparable to the size of cells. An application-specific printed circuit board (PCB) was designed for the implementation of the impedance measurement in order to facilitate connection with the device used for taking EIS spectra and for selecting the channels to be measured. The PCB was designed in consideration of the optical screening of trapped cells in parallel with the EIS measurements which allows the comparison of EIS data with optical signals. With continuous EIS measurement, the filling of channels with cell suspension can be followed. Yeast cells were trapped in the microfluidic system and EIS spectra were recorded considering each individual channel, which allows differentiating between the number of trapped cells.

## 1. Introduction

The application of Lab-on-chip and Organ-on-Chip devices, designed for the in vitro study of cell populations or even single cells in a specific chemical environment, can be a significant step forward in drug discovery, screening and developing therapeutic strategies [1,2,3]. Application-specific devices integrating microfluidic and sensing systems are capable of simultaneously maintaining artificial cell populations and tissues, treating them with chemical agents, and monitoring their physiological behavior in real-time [4]. This multifunctional behavior is crucial in personalized medicine and drug development [5,6]. Impedance spectroscopy can be used to characterize different properties of cells and cell cultures, as it allows non-invasive and real-time measurements [7]. In recent years there has been a huge rise in the development of microfluidic devices that integrate electrodes for Electrochemical impedance spectroscopy (EIS) in order to create versatile biosensors [8]. Using integrated microelectrodes, microfluidic systems can be adapted to perform impedance-based cell analysis [7] to determine confluency and to quantify cell number [9], to measure response to external stimuli [10], to trap cells and monitor their growth [11] or to detect bacteria [12]. There are many applications using flow cytometry combined with impedance sensing that aim to study general cell status [13,14]. This method builds on fluid flowing through the channels and the underlying electrodes for fast characterization and selection of a vast number of cells; however, it is inappropriate for studying individual cells for a longer period of time [15]. By incorporating cell trapping sites into the microchips, the prolonged behavior of single cells can be studied in a stable environment, like continuous monitoring of a single HeLa cell [16], to track the individual metabolism of bacteria [17] or to examine single-cells with impedance tomography methods [18].

3D cell culture methods offer numerous advantages over 2D cell cultures like improved drug metabolism and more accurate representation; however, they remain expensive and the experiments are difficult to replicate and to interpret [19]. For this reason, the application of 2D cell culture methods is still relevant for long-term experiments and for the initial testing of a designed system [20].

Impedance (*Z*) is the alternating current (AC) analogy of the resistance in direct current (DC) applications defined by the following:

(1) $$Z\left(f\right)=\frac{V(f)}{I(f)}=Z^{\prime }+i\cdot Z^{\prime \prime }=\left|Z\right|e^{i\phi }$$

where both the voltage (*V*) and the current (*I*) are dependent on the applied frequency (*f*). Impedance is a complex quantity, the real part of the impedance ($Z^{\prime }$) describes the resistive properties while the imaginary part ($Z^{\prime \prime }$) describes the capacitive and inductive properties of the system. The polar form of the equation includes the absolute value of the impedance (|Z|) and the phase (ϕ). Conducting impedance measurements on a wide range of frequencies (10 kHz–1 MHz) creates the impedance spectrum which can provide insight into the inner structure of a live organism. The measurements can be represented on either a Nyquist or a Bode plot to visualize the frequency-dependent behavior of the system owing to the different elements that make up the electrochemical system. Equivalent circuit fitting is generally used in electrochemical and cellular applications in order to model the system and to determine the electrical parameters that describe its different physical and chemical properties [21]. The basic elements that the equivalent circuit consists of can be resistances (media), capacitors (membranes, double layers), or inductances, connected in series and in parallel. Other specific elements can be used, for example, constant phase elements (CPE) to describe the non-ideal behavior of capacitors or Warburg elements which describe diffusional processes [22].

In biological applications, a wide range of frequencies from 1 Hz to 10 GHz is used in EIS measurements depending on the electrical and cellular components that need to be studied [23]. Three characteristic regions were distinguished within the frequency spectrum used for AC signals, namely α, β and γ dispersions [24]. In the α-dispersion range (below a few kHz) the polarization of the electrodes dominates the spectrum. The β-dispersion (few kHz to 1 GHz) arises from the effects of the plasma membrane. This range can be divided into two domains: from 1 to 10 MHz and from 10 MHz to 1 GHz [25]. The lower region corresponds to the charging between the plasma membrane of cells and the surrounding medium. In the latter region where the frequency is sufficiently high the cell membrane is short-circuited and charging occurs between the cytoplasm and the surrounding medium. The γ-dispersion is above 1 GHz and refers to the reorientation of water molecules. The β-dispersion region is appropriate for studying cellular properties like cell size and features of the cell membrane or the cytoplasm. During the measurements the combined range of α and β dispersions (10 Hz–1 MHz) was used to study both cellular and electrode responses.

Individual examination and characterization of cells by optical and electrical methods can be a powerful combination for observing the changes occurring in cellular properties due to various effects. The initial testing included differentiation of the cell numbers to present that the device can potentially be used to monitor cell proliferation in microenvironments. This method aims to eventually forgo the optical feedback that generally needs labeling for fluorescence. The used dyes can modify cellular behavior [26,27,28], thus creating a purely electric non-invasive measurement method can prove beneficial in various applications.

In this work, a microfluidic device with integrated microelectrodes was created which can trap cell populations or even individual cells between the sensing electrodes. The system is designed to simultaneously measure the impedance spectra with continuous, real-time optical feedback to characterize the changes that localized cells cause in their microenvironment. The proof of the concept of the method was realized by using yeast cells (*S. cerevisiae*) due to their many advantageous qualities like fast division and ease of cultivation [29].

## 2. Materials and Methods

### 2.1. Materials

Polydimethylsiloxane (PDMS), Dow Sylgard 184 kit was used for soft lithography and modified by Poly(dimethylsiloxane-b-ethylene oxide) (PDMS-b-PEO) purchased from Polysciences Europe GmbH (Eppelheim, Germany). *S. cerevisiae* yeast cells and phosphate-buffered saline (PBS) were purchased from Sigma-Aldrich Ltd. (Burlington, MA, USA). Propidium Iodide ReadyProbes were purchased from Thermo Fisher Scientific Inc. (Waltham, MA, USA).

### 2.2. Microbial Cultivation

*S. cerevisiae* yeast cells from Sigma–Aldrich Ltd. were grown in a pH 7.4 PBS buffer solution and were further diluted with PBS to obtain the desired $10^{6}$ cell/mL concentration for trapping and for fluorescent staining. Viability testing was achieved by propidium-iodide staining according to the protocol of the used Propidium Iodide ReadyProbes from Thermo Fisher. By adding two drops of the reagent per mL to the solution containing the recommended $10^{6}$ cells/mL, an adequate fluorescent signal was achieved. The viability of the cells was checked after 15 min in a NanoEntek C-Chip 4ch hemacytometer with fluorescent microscopy, and the viable cell cultures were further used in the microfluidic device.

### 2.3. Device Fabrication

**Figure 1:**
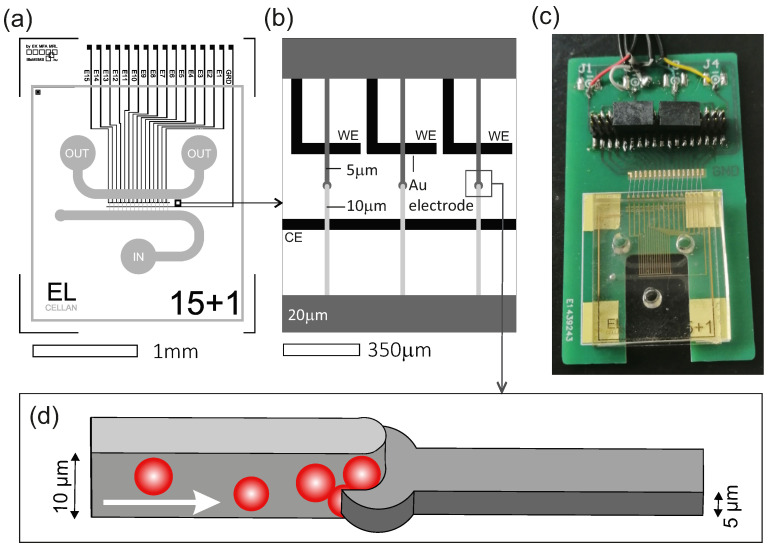
(**a**) Layout of the microfluidic chip (light grey) and the underlying electrode system (black). (**b**) Closeup on the trapping cross-channels and the electrodes. (**c**) The designed and manufactured PCB contains a window for optical screening and the assembled microfluidic device. (**d**) Schematic figure of the trapping site showing how cells are caught in the vertically narrowing channel.

The layout of the microfluidic system was inspired by the microfluidic device used by Taylor et al. in axon and neuron growth studies, although significant modifications were applied with regard to the integration of the cell trapping region and sensing electrodes [30]. The microfluidic structure consists of two main channels which are connected by 15 smaller cross-channels that narrow vertically, thus enabling cell trapping. The chip has an inlet and a small venting valve to ensure proper filling and fluid flow toward the two outlets. The electrodes were designed with the ability to monitor each channel individually in mind. There is a common counter electrode (CE) running along the length of the channels and individual working electrodes (WE) go towards each channel. The layout of the microfluidic structure and the electrodes are shown in Figure 1.

In order to perform impedance spectroscopy analysis in the microfluidic chip, electrodes were designed to lay under each channel and were integrated into the microfluidic cell. In addition, a PCB was designed and fabricated that allows measurements on the electrodes to be performed in parallel on three channels (Figure 1). Individual or multiple channels can be also selected for analysis. The PCB with the assembled structure can be connected to a PalmSense4 potentiostat (PalmSens BV, Houten, The Netherlands), which is capable of performing impedance spectroscopy from 10 µHz to 1 MHz. Data evaluation and circuit fitting can be achieved with the associated controlling and analyzing software (PSTrace 5.8). A window in the bottom center of the PCB provides insight into the channels and allows optical microscopy in parallel with the EIS measurements.

The designed electrode arrangement allows recording of the impedance spectrum of individual channels. Cell trapping between the two electrodes causes changes in the characteristic impedance of the system which enables the deduction of cellular numbers and biophysical properties (size, viability, etc.). The electrodes have been prepared by vacuum evaporation of 150 nm thick gold layer onto the Borofloat glass (Schott AG, Mainz, Germany) substrate applying 5 nm titanium adhesive layer and patterned by lift-off lithography. The width of the electrodes is 50 µm and their layout was designed to ease the alignment of the microfluidic channels. The electrodes are covered with a SU-8 photoresist as an insulating layer (Kayaku Advanced Materials, Inc., Westborough, MA, USA) with the corresponding contact windows remaining open. The distance between the CE and WE is 300 µm which enables aligning the electrodes precisely to encompass the traps in the channels with a microscope.

The microfluidic chip was fabricated by standard soft-lithography and replica molding in PDMS [31]. The master mold was prepared on a 4″ silicon wafer by three-step photolithography using SU-8 2005, 2010 and 2025 photoresists subsequently for creating different heights for the channels according to their functions. The positive relief contained 12 structures and was cast with 1:10 PDMS containing 1:200 PDMS-b-PEO to render the channel surfaces hydrophilic. The mold with the PDMS was cured at 65 °C for 2 h. After heat treatment, the chips were cut and peeled off from the mold. Every chip was individually cleaned in an ultrasonic shaking bath in deionized water and in isopropyl alcohol. Then the chips were manually aligned with the electrodes under a microscope to ensure proper positioning of the vertically narrowing cell trapping channels.

The size of the chip is 20 × 18 mm^2^, the height of the main channels are 20 µm and their widths are 800 µm, the cross-channels narrow vertically from 10 µm to 5 µm and their widths are 20 µm. The diameter of the inlets and outlets is 3000 µm with adequate holes punched for filling the channels.

## 3. Results and Discussion

### 3.1. Detection of Filling the Cell Traps

**Figure 2:**
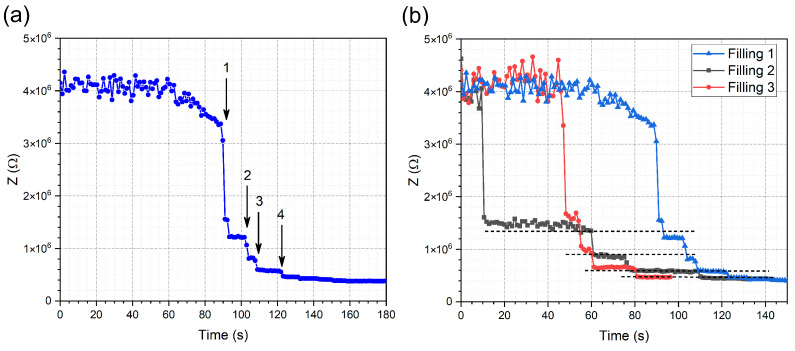
The step-like decreases in impedance when fluid fills the channels and connects the parallel electrodes (**a**). Arrows indicate the steps caused by the fluid sequentially flowing over each of the four individual electrode pairs, thus increasing the conductivity of the system. Repeated fillings show the stability of the characteristic impedance values indicated by dashed lines (**b**).

To monitor the loading characteristics of the device 20 µL of 0.01 M PBS solution was used to prime the channels and the underlying electrodes. To detect the proper filling of the channels, the time-dependent EIS spectrum of four parallel Au electrodes was monitored. At a low frequency, the spectrum is dominated by the electrode characteristics the impedance was measured at a fixed 1 kHz frequency [25]. As the buffer solution reaches and connects, the electrodes’ step-like decreases can be observed in the impedance due to the specific conductivity of the media (Figure 2). The mentioned steps indicate the proper filling of the microfluidic system and the adequate electrode response which can be applied for preliminary characterization and the properties of the electrodes and the used solution. The value of the steps decrease with 1/Z as the parallel electrodes are being electrically connected. Figure 2b shows three repeated fillings to demonstrate the repeatability of the measurements, the dashed lines indicate the average values of the stabilized impedance achieved after filling a channel. Table 1 shows the corresponding average impedance of the different fillings and their standard deviation. At first Z is very high because the system is open-circuited, and when the fluid reaches and starts to fill the electrode surface with the solution, there is a slight decrease followed by the first step. After that, as the solution reaches the other electrodes additional steps appear in the spectrum.

### 3.2. Concentration and pH Dependence

**Figure 3:**
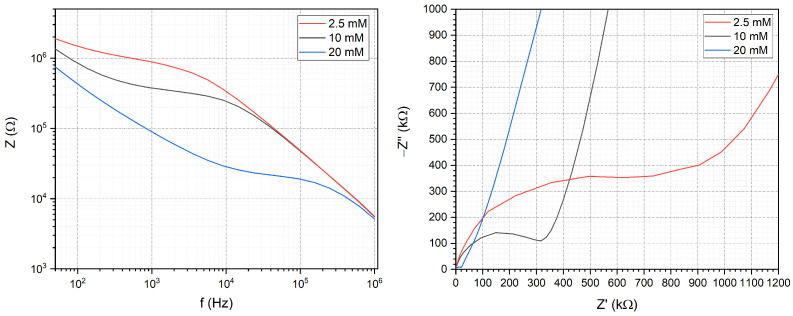
Concentration dependence of the impedance spectra measured in different dilutions of PBS. The Bode (**left**) and Nyquist (**right**) plots show the difference in conductivity of the dilutions.

**Figure 4:**
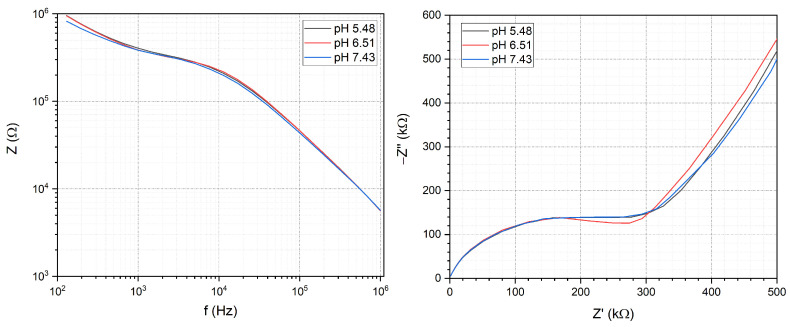
Investigation of pH dependence for PBS with different pHs shown on Bode (**left**) and Nyquist (**right**) plots. The Au electrodes show no significant pH dependence.

In order to functionally validate the impedance measurement system PBS solutions with different concentrations and different pHs were injected into the microfluidic channels. The solutions were prepared by dissolving PBS tablets (Sigma-Aldrich) in 100 mL, 200 mL and 800 mL of deionized water which yields 20 mM and 10 mM and 2.5 mM concentrations, respectively. The EIS spectra of the solutions were taken between 1 Hz and 1 MHz and are shown in Figure 3 both in Bode and Nyquist diagrams. As the concentration of the solution increases, the measured impedance decreases due to the increased conductivity of the system. The decrease can be seen on the Bode plot on the magnitude of the impedance and on the Nyquist plot by the decrease in the radius of the circular functions belonging to the different concentrations. The pH dependence was investigated with 0.01 M PBS solution on pH 5.48, pH 6.51 and pH 7.43. The impedance of Au electrodes shows no significant pH sensitivity during the EIS measurements as represented in Figure 4.

### 3.3. Reroducibility and Stability

**Figure 5:**
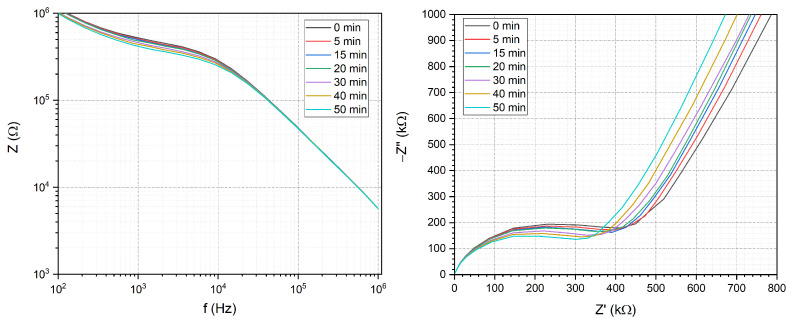
The stability of the system was investigated for 50 min continuously. There is a slight decrease in impedance due to the evaporation of the solution as represented by the Bode (**left**) and Nyquist (**right**) plots.

**Figure 6:**
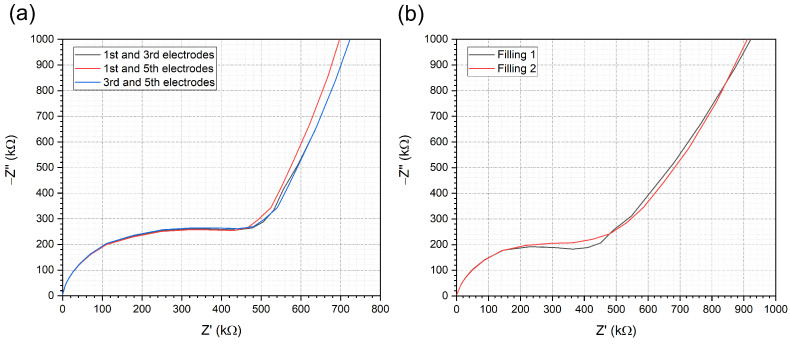
(**a**) Comparison of the impedance spectra measured on electrode pairs characterising different parallel microfluidic channels by connecting the first (1), third (3) and fifth (5) channels. The slight effect of cleaning and refilling the microfluidic channels demonstrates the repeated usability of the proposed system (**b**).

To examine the reproducibility and the stability of the system, measurements were performed with repeated filling while observing the EIS spectrum for a longer period of time. The EIS spectra were recorded with 5–10 min time differences (Figure 5). The impedance spectra slightly change in the low-frequency region (under $10^{4}$ Hz) in the first 10–15 min, due to the stabilization of the electrical properties of the system. With time there is a slow decrease in impedance owing to the evaporation of solution from the microfluidic chip; otherwise, the system is stable within a maximum of 20% deviations on the time range of the standard measurements and up to 10% under 30 min. A comparison of EIS spectra of three different electrodes yielded stable results. Here, the first, third and fifth electrodes were each coupled and their EIS spectra were examined. Then, the capability for repeated usage was investigated by measuring the the same chip after direct refilling (Figure 6). In both cases, the EIS spectra show reproducibility.

### 3.4. EIS Analysis of Localised Cells

**Figure 7:**
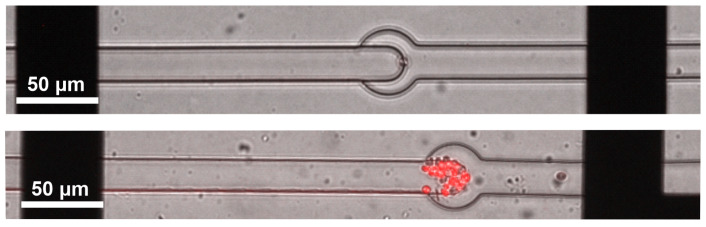
A single yeast cell trapped between the measuring electrodes (**top**) and the propidium-iodide staining showing the viability of the cells (**bottom**).

To validate the ability of the analytical system to screen localized cells in the microfluidic structure, *S. cerevisiae* yeast cells were trapped in the channels and their effects on the EIS spectrum were characterized. A yeast solution was prepared in PBS with 0.05 mg/mL concentration. This concentration yields around $10^{6}$ yeast cells per mL which is the optimal concentration to trap individual cells in the narrowing microfluidic channels (Figure 7). The number of successfully trapped cells was identified with a Zeiss AxioVert A1 inverted fluorescent microscope equipped with a Zeiss Axiocam 506 mono microscope camera (Zeiss, Oberkochen, Germany) connected to the Zeiss ZEN 2.6 (blue edition) image processing software. Propidium-iodide staining was applied to follow the changes in the viability of the cells (Figure 7) which was monitored by fluorescent microscopy using Cy3 fluorescent filter set (ex. 554 nm, em. 566 nm) for illumination and detection.

**Figure 8:**
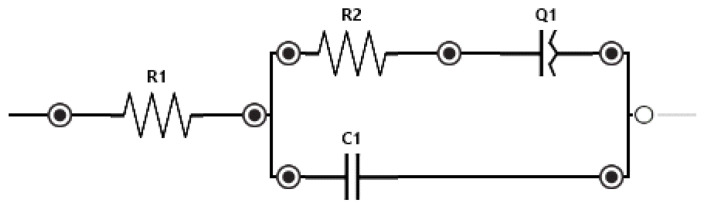
The equivalent circuit elements of a modified Randles cell substituting the Warburg element by CPE (Q) (**left**) and the cell number dependency of $R_{ct}$ fitted to the measured EIS spectra (**right**). The table contains the values for the individual (**top half**) and the three parallel (**bottom half**) electrodes, as well as the errors coming from the fitting.

**Figure 9:**
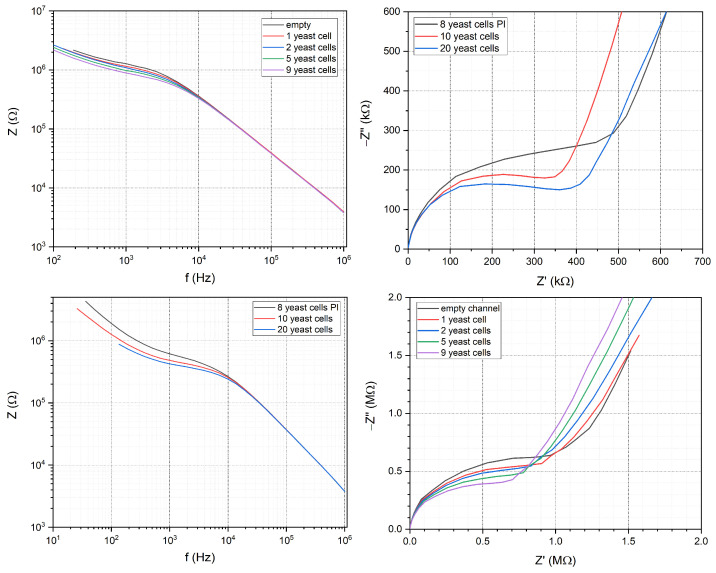
Bode (**left**) and Nyquist (**right**) plots of different numbers of trapped cells between individual (**top**) and three parallel (**bottom**) gold electrodes.

EIS measurements were performed to reveal the effect of the cells on the characteristic impedance spectrum and to differentiate between the number of successfully trapped cells. Model fitting was accomplished by fitting the Randles equivalent circuit to the data with the software (PSTrace 5.8) provided by the PalmSense4 potentiostat that was used to record the impedance spectra. The model was modified by substituting the Warburg element, which normally describes the diffusional behavior of the system at low frequencies with a 45° line on the Nyquist plot, with a CPE accounting for the additional capacitive behavior which yielded a ∼70° line in the low-frequency region (Figure 8). Measurements were implemented by either using individual or three parallel electrodes connected to characterize cell trapping. Cell trapping happens stochastically in the system during filling which yields different cell numbers between the electrodes. The trapping is followed optically with a microscope and when the system stabilizes and the cells are trapped in the vertically narrowing channels, the EIS spectrum is recorded on the selected electrodes. The difference between the EIS spectra of 0 (empty trap), 1, 2, 5 and 9 trapped yeast cells was measured on individual electrodes. Three electrodes were used to record the impedance of 8, 10 and 20 trapped yeast cells shown on Figure 9. These numbers are the sum of the trapped cells between the three electrodes used during the measurement.

The recorded EIS spectrum was fitted with the described Randles cells and the corresponding parameters were derived which are the resistance of the (extracellular) solution ($R_{s}$), the charge transfer resistance ($R_{ct}$), the double layer capacitance ($C_{dl}$) and the CPE (Q). The experienced effect of the trapped yeast cells on the impedance spectra is in good agreement with the literature [32]. Apart from the mentioned parameters, the cell membrane capacitance ($C_{m}$) can also characterize the state of cells at high frequencies [14,33,34]. However, in our measurement setup we do not have the appropriate frequency range for examining the influence of $C_{m}$ to the spectra. The characteristic parameter that significantly changes when different numbers of cells are trapped between the electrodes is $R_{ct}$. This charge transfer resistance corresponds to the transmembrane ion exchange between the solution and the cell cytoplasm increasing the conductivity of the electrical double layer on the electrode surface by ion adsorption [32,35]. The fitted values and the errors of the fitting for $R_{ct}$ in both cases are shown in Table 2. The results using individual electrodes show close to linear behavior in $R_{ct}$ values; however, the measurements were hindered by the fact that during the filling protocol, we have no accurate control over how many cells are trapped in which of the channels. In the case of the connected electrode configuration, the offset of charge transfer resistance was decreased due to the parallel microfluidic channels. The modified Warburg impedance (CPE) was elevated due to the increased electrode surface—compared to the single electrode system. In the case of the modified solution composition (additional glucose and propidium-iodide staining when eight cells were analyzed) in Figure 9 a slight change in the solution resistance ($R_{s}$) was experienced.

## 4. Conclusions

A cell analytical microfluidic system was designed and fabricated with underlying electrodes to trap cells and simultaneously monitor their characteristics with electrochemical impedance spectroscopy. The system was proved to be applicable for screening localized cells by the non-invasive, label-free EIS method that is ideal for physiological cell analysis. A dedicated PCB was also designed for the measurement to enable the selection of individual or multiple channels to study the different effects during cell trapping. Continuous EIS measurement on a fixed 1 kHz frequency was used for tracking the sample injection into the channels and for initial functional testing of the electrodes. Yeast cells were trapped in the vertically narrowing channels between the electrodes and were analyzed by EIS spectroscopy. Modified Randles equivalent circuit model was fitted to derive characteristic parameters of the cell-containing system. The charge transfer resistance was identified as a characteristic parameter of the circuit model directly correlating to the number of localized cells. The cell number dependency of the charge transfer resistance originates in the ion exchange between the extracellular solution and the cytoplasm increasing with the cell number.

The proposed microfluidic system offers possibilities for monitoring individual cells or cell populations. Following the growth and viability of cells could be crucial in drug efficiency and susceptibility testing. Future work will include implementing this technique with a more robust application with different cell types using cell-specific microfluidic trapping systems, and the possible applications for microfluidic impedance tomography methods.

## Figures and Tables

**Table 1:** The averaged impedance values of the filling steps and their standard deviation.

Steps	Filling 1 (Ω)	Filling 2 (Ω)	Filling 3 (Ω)	Average (Ω)
1	1.46 $\times \;10^{6}$ $\pm \;6.21\times 10^{4}$	1.26 $\times \;10^{6}$ $\pm \;1.48\times 10^{5}$	1.37 $\times \;10^{6}$ $\pm \;3.19\times 10^{5}$	1.37 $\times \;10^{6}$ $\pm \;1.00\times 10^{5}$
2	8.66 $\times \;10^{5}$ $\pm \;2.25\times 10^{4}$	8.04 $\times \;10^{5}$ $\pm \;2.54\times 10^{4}$	9.89 $\times \;10^{5}$ $\pm \;4.93\times 10^{4}$	8.86 $\times \;10^{5}$ $\pm \;9.44\times 10^{4}$
3	5.91 $\times \;10^{5}$ $\pm \;3.23\times 10^{4}$	5.77 $\times \;10^{5}$ $\pm \;1.12\times 10^{4}$	6.53 $\times \;10^{5}$ $\pm \;8.74\times 10^{3}$	6.07 $\times \;10^{5}$ $\pm \;4.09\times 10^{4}$
4	4.44 $\times \;10^{5}$ $\pm \;9.77\times 10^{3}$	3.98 $\times \;10^{5}$ $\pm \;2.61\times 10^{4}$	4.68 $\times \;10^{5}$ $\pm \;3.35\times 10^{3}$	4.37 $\times 10^{5}$ $\pm \;3.56\times 10^{4}$

**Table 2:** The number of trapped cells and the fitted charge transfer resistance values ($R_{ct}$) with the errors of the fitting for the individual (**top half**) and the three parallel (**bottom half**) electrodes.

Cell Number	$R_{\mathrm{ct}}$ [kΩ]
0	1106 ± 6.91%
1	1009 ± 7.70%
2	898.8 ± 4.44%
5	814.8 ± 4.34%
9	758.4 ± 2.72%
8	466.1 ± 3.99%
10	405.4 ± 2.72%
20	349.5 ± 3.44%

## Data Availability

Data are contained within the article.

## Abbreviations

The following abbreviations are used in this manuscript:

CE	Counter Electrode
CPE	Constant Phase Element
EIS	Electrochemical Impedance Spectroscopy
PCB	Printed Circuit Board
WE	Working Electrode
